# Telomere length analysis in amyotrophic lateral sclerosis using large-scale whole genome sequence data

**DOI:** 10.3389/fncel.2022.1050596

**Published:** 2022-12-15

**Authors:** Ahmad Al Khleifat, Alfredo Iacoangeli, Ashley R. Jones, Joke J. F. A. van Vugt, Matthieu Moisse, Aleksey Shatunov, Ramona A. J. Zwamborn, Rick A. A. van der Spek, Johnathan Cooper-Knock, Simon Topp, Wouter van Rheenen, Brendan Kenna, Kristel R. Van Eijk, Kevin Kenna, Ross Byrne, Victoria López, Sarah Opie-Martin, Atay Vural, Yolanda Campos, Markus Weber, Bradley Smith, Isabella Fogh, Vincenzo Silani, Karen E. Morrison, Richard Dobson, Michael A. van Es, Russell L. McLaughlin, Patrick Vourc’h, Adriano Chio, Philippe Corcia, Mamede de Carvalho, Marc Gotkine, Monica Povedano Panades, Jesus S. Mora, Pamela J. Shaw, John E. Landers, Jonathan D. Glass, Christopher E. Shaw, Nazli Basak, Orla Hardiman, Wim Robberecht, Philip Van Damme, Leonard H. van den Berg, Jan H. Veldink, Ammar Al-Chalabi

**Affiliations:** ^1^Department of Basic and Clinical Neuroscience, Maurice Wohl Clinical Neuroscience Institute, King’s College London, London, United Kingdom; ^2^Department of Biostatistics and Health Informatics, Institute of Psychiatry, Psychology and Neuroscience, King’s College London, London, United Kingdom; ^3^Department of Neurology, University Medical Center (UMC) Utrecht Brain Center, Utrecht University, Utrecht, Netherlands; ^4^Department of Neurosciences, Experimental Neurology, KU Leuven—University of Leuven, Leuven, Belgium; ^5^VIB Center for Brain & Disease Research, Laboratory of Neurobiology, Leuven, Belgium; ^6^Institute of Medicine, North-Eastern Federal University, Yakutsk, Russia; ^7^Department of Molecular and Clinical Pharmacology, University of Liverpool, Liverpool, United Kingdom; ^8^Sheffield Institute for Translational Neuroscience (SITraN), University of Sheffield, Sheffield, United Kingdom; ^9^Complex Trait Genomics Laboratory, Smurfit Institute of Genetics, Trinity College Dublin, Dublin, Ireland; ^10^Computational Biology Unit, Instituto de Salud Carlos III, Madrid, Spain; ^11^School of Medicine, Translational Medicine Research Center-NDAL, Koc University, Istanbul, Turkey; ^12^Neuromuscular Diseases Unit/ALS Clinic, Kantonsspital St. Gallen, St. Gallen, Switzerland; ^13^Department of Neurology and Laboratory of Neuroscience, IRCCS Istituto Auxologico Italiano, Milan, Italy; ^14^Department of Pathophysiology and Transplantation, “Dino Ferrari” Center, Università degli Studi di Milano, Milan, Italy; ^15^Faculty of Medicine, Health and Life Sciences, Queen’s University Belfast, Belfast, United Kingdom; ^16^Institute of Health Informatics, University College London, London, United Kingdom; ^17^Centre SLA, CHRU de Tours, Tours, France; ^18^Department of Neuroscience, ALS Centre, University of Torino, Turin, Italy; ^19^Azienda Ospedaliera Citta della Salute e della Scienza, Turin, Italy; ^20^Federation des Centres SLA Tours and Limoges, LITORALS, Tours, France; ^21^Physiology Institute, Faculty of Medicine, Instituto de Medicina Molecular, University of Lisbon, Lisbon, Portugal; ^22^Department of Neurology, Hadassah Medical Organization and Faculty of Medicine, Hebrew University of Jerusalem, Jerusalem, Israel; ^23^Department of Neurology, Hospital Universitari de Bellvitge, Barcelona, Spain; ^24^Hospital San Rafael, Madrid, Spain; ^25^Department of Neurology, University of Massachusetts Medical School, Worcester, MA, United States; ^26^Department of Neurology, Center for Neurodegenerative Diseases, Emory University, Atlanta, GA, United States; ^27^King’s College Hospital, London, United Kingdom; ^28^Academic Unit of Neurology, Trinity Biomedical Sciences Institute, Trinity College Dublin, Dublin, Ireland; ^29^Department of Neurology, Beaumont Hospital, Dublin, Ireland; ^30^Department of Neurology, University Hospitals Leuven, Leuven, Belgium

**Keywords:** amyotrophic lateral sclerosis (ALS), telomere–genetics, whole genome sequence (WGS), genomics, bigdata, MND–motor neuron disorders

## Abstract

**Background:**

Amyotrophic lateral sclerosis (ALS) is a neurodegenerative disease characterized by the loss of upper and lower motor neurons, leading to progressive weakness of voluntary muscles, with death following from neuromuscular respiratory failure, typically within 3 to 5 years. There is a strong genetic contribution to ALS risk. In 10% or more, a family history of ALS or frontotemporal dementia is obtained, and the Mendelian genes responsible for ALS in such families have now been identified in about 50% of cases. Only about 14% of apparently sporadic ALS is explained by known genetic variation, suggesting that other forms of genetic variation are important. Telomeres maintain DNA integrity during cellular replication, differ between sexes, and shorten naturally with age. Sex and age are risk factors for ALS and we therefore investigated telomere length in ALS.

**Methods:**

Samples were from Project MinE, an international ALS whole genome sequencing consortium that includes phenotype data. For validation we used donated brain samples from motor cortex from people with ALS and controls. Ancestry and relatedness were evaluated by principal components analysis and relationship matrices of DNA microarray data. Whole genome sequence data were from Illumina HiSeq platforms and aligned using the Isaac pipeline. TelSeq was used to quantify telomere length using whole genome sequence data. We tested the association of telomere length with ALS and ALS survival using Cox regression.

**Results:**

There were 6,580 whole genome sequences, reducing to 6,195 samples (4,315 from people with ALS and 1,880 controls) after quality control, and 159 brain samples (106 ALS, 53 controls). Accounting for age and sex, there was a 20% (95% CI 14%, 25%) increase of telomere length in people with ALS compared to controls (p = 1.1 × 10^−12^), validated in the brain samples (p = 0.03). Those with shorter telomeres had a 10% increase in median survival (p = 5.0×10^−7^). Although there was no difference in telomere length between sporadic ALS and familial ALS (p=0.64), telomere length in 334 people with ALS due to expanded *C9orf72* repeats was shorter than in those without expanded *C9orf72* repeats (p = 5.0×10^−4^).

**Discussion:**

Although telomeres shorten with age, longer telomeres are a risk factor for ALS and worsen prognosis. Longer telomeres are associated with ALS.

## Introduction

Amyotrophic lateral sclerosis (ALS) is a neurodegenerative disease affecting motor neurons in the brain and spinal cord resulting in progressive paralysis and death, within three to five years, typically due to respiratory failure (Brown and Al-Chalabi, 2017; Hardiman et al., 2017). The first symptoms of weakness can occur in the bulbar innervated muscles, manifesting as difficulty with speech or swallowing, or in the spinal innervated muscles, manifesting as limb weakness or breathing difficulty. About 5% may have a frank frontotemporal dementia, and frontotemporal impairment is seen in up to 80% of people by the time King’s Stage 4 disease is reached (Roche et al., 2012; Crockford et al., 2018).

The last decade has seen substantial advances in our understanding of the genomic basis of ALS (van Rheenen et al., 2021; Hop et al., 2022) but a significant proportion of the genetic contribution to risk remains unexplained. This hidden heritability may be harbored in other types of genomic variation as well as in rare variants that may be unique to an affected individual or family (Al-Chalabi et al., 2010; McLaughlin et al., 2015).

Telomeres are repeated TTAGGG nucleotide sequences located at the ends of chromosomes and exist to maintain chromosomal structural integrity during cellular replication. They shorten naturally with age and differ in average length between the sexes (Muzumdar and Atzmon, 2012; Kong et al., 2013; Gardner et al., 2014); age and sex are also risk factors for ALS (McCombe and Henderson, 2010; Al-Chalabi and Hardiman, 2013; Westeneng et al., 2018). Telomere length is a marker for aging, chromosomal instability and DNA damage, and might therefore be relevant as a risk factor for ALS (Murnane, 2006; Conomos et al., 2012; Xu et al., 2013; Marzec et al., 2015).

Previously, in a pilot study in a UK cohort, we showed that longer telomeres might be associated with ALS when compared to age and sex-matched controls (Al Khleifat et al., 2019). We therefore sought to explore this finding in detail, using whole-genome sequence data from the Project MinE consortium (van Rheenen et al., 2018), a large international ALS genomics collaboration.

## Materials and methods

### Data sources and data extraction

#### Blood samples

Samples were from the international Project MinE whole genome sequencing consortium and derived from seven countries: the USA, Ireland, Belgium, the Netherlands, Spain, Turkey, and the United Kingdom (van Rheenen et al., 2018).

DNA was isolated from venous blood using standard methods. The DNA concentrations were set at 100 ng/ul as measured by a fluorimeter with the PicoGreen^®^ dsDNA quantitation assay. DNA integrity was assessed using gel electrophoresis.

### Post-mortem samples

Post-mortem motor cortex was from the MRC London Neurodegenerative Diseases Brain Bank based at the Institute of Psychiatry, Psychology and Neuroscience, King’s College London. Tissue was flash frozen stored at −80^°^C. 100 mg tissue blocks were excised. DNA was isolated from the same tissue block and sequenced. The study cohort consisted of 64 people with apparently sporadic ALS and 53 controls with no known neurological disease (controls below Hyperphosphorylated tau (HP-τ) in human brain tissue and BNE\Braak stage 2).

The 100 mg tissue blocks were divided to allow DNA purification. For each sample, a 25 mg tissue block for DNA was homogenized using a Qiagen PowerLyzer 24 Homogenizer. DNA was purified from the homogenate using the standard protocol from Qiagen’s DNeasy Blood and Tissue Mini Kit. DNA was quantified using PicoGreen (Quant-iT™ PicoGreen^®^ dsDNA Reagent, ThermoFisher Scientific) and measured using a Spectromax Gemini XPS (Molecular Devices).

### Library preparation and DNA sequencing

Library preparation was performed using the Illumina DNA Sample Preparation HT Kit alongside the Illumina SeqLab DNA PCR-Free Library Prep Guide. Libraries were then quantified using qPCR and evaluated using gel electrophoresis. All samples were sequenced using Illumina’s FastTrack services (San Diego, CA, USA). Some blood-derived DNA samples were sequenced using the Illumina HiSeq 2000 platform. Sequencing was 100 bp paired-end performed using PCR-free library preparations and targeted ∼40x coverage across each sample. Remaining blood-derived and all brain-derived DNA libraries were clustered onto flow cells using the Illumina cBot System, as per cBot System Guide using Illumina HiSeq X HD Paired End Cluster Kit reagents and sequenced on an Illumina HiSeqX with 151 bp paired-end runs using independent flow cell lanes and with a target minimum of 30x average coverage per sample. Binary sequence alignment/map formats (BAM) were generated for each individual. All the genomes were aligned with Isaac (Illumina) to hg19. The details of the Isaac alignment and variant calling pipelines are discussed in Project MinE design (van Rheenen et al., 2018) and the Isaac protocol (Raczy et al., 2013).

### Determination of telomere length

TelSeq (Ding et al., 2014) was used to quantify telomere length using whole genome sequence data. Telomere lengths were estimated from reads, defined as repeats of more than seven TTAGGG motifs.

### Statistical analysis

The effect of telomere length on ALS risk was tested using a multivariable linear regression model. To account for different sequencing platforms and population stratification, principal components of ancestry, center and technology platform were included as covariates. To assess the model, Pearson’s chi-squared test was used. Because telomere length correlates with age, we performed an additional test to examine the possibility that survival bias could affect the results. To do this, we also performed the analysis restricted to the subgroup of people with ALS onset below the median cohort age (62 years). As brain is composed of neurons which do not divide, as well as glia which do, we expected that the average telomere length in brain would be longer than in blood. Furthermore, nervous tissue is the target of the disease process rather than blood. We therefore additionally tested the effect of age on telomere length in brain tissue.

To determine if telomeres are lengthened in ALS, or simply shorten less rapidly than in controls, we analysed the effect of age on telomere length in each group using multivariable linear regression.

To assess the effect of covariates on telomere length affecting survival, we used Cox regression, controlling for age, sex and site of disease onset (bulbar or spinal), population stratification, principal components, center and sequencing platforms.

Repeat primed PCR and Expansion Hunter-v2.5.1 (Dolzhenko et al., 2017) were used to assay the hexanucleotide repeat expansion in the *C9orf72* gene since this is a known risk factor for ALS and associates with survival.

Statistical tests were performed using IBM SPSS Statistics 24.0 (SPSS Inc., Armonk, NY, USA), and RStudio, R Foundation for Statistical Computing 3.4.1 (RStudio, Vienna, Austria).

### Quality control

Quality control was performed separately on the genotyped data of each population as reported previously (van Rheenen et al., 2016; Supplementary Appendix 1).

### Ethical approval

Informed consent was obtained from all participants in this project according to the ethical approval at each participating Project MinE site as previously described (van Rheenen et al., 2018).

## Results

There were 6,580 whole genome sequences (4,515 from people with ALS and 2,065 from controls), reducing to 6,195 [4,315 (95.6%) from people with ALS and 1,880 (91%) from controls] after quality control, with minimum ∼25x coverage across each sample. The set was enriched for apparently sporadic ALS [4,236 compared with 79 with familial ALS (FALS)]. The male-female ratio was 2:1. Overall, 22 had ALS-frontotemporal dementia (ALS-FTD). Phenotypically, 37 had pure progressive bulbar palsy (PBP) and 68 and progressive muscular atrophy. There were 1,908 sequenced using the HiSeq2000 platform and 4,287 sequenced using the HiSeqX Illumina platform (Table 1). There were 344 people carrying an expanded *C9orf72* hexanucleotide repeat, 334 with ALS and 10 without symptoms.

**TABLE 1:** Detailed demographic features of the study population.

Cohort	Sample	Case	Control	Female	Male
Belgium	548	368	180	209	339
Ireland	403	267	136	161	242
Netherlands	2894	1859	1035	1182	1712
Spain	338	233	105	145	193
Turkey	223	148	75	87	136
United Kingdom	1402	1124	278	603	799
United States	387	316	71	153	234
Total	6195	4315	1880	2540	3655

**FIGURE 1:**
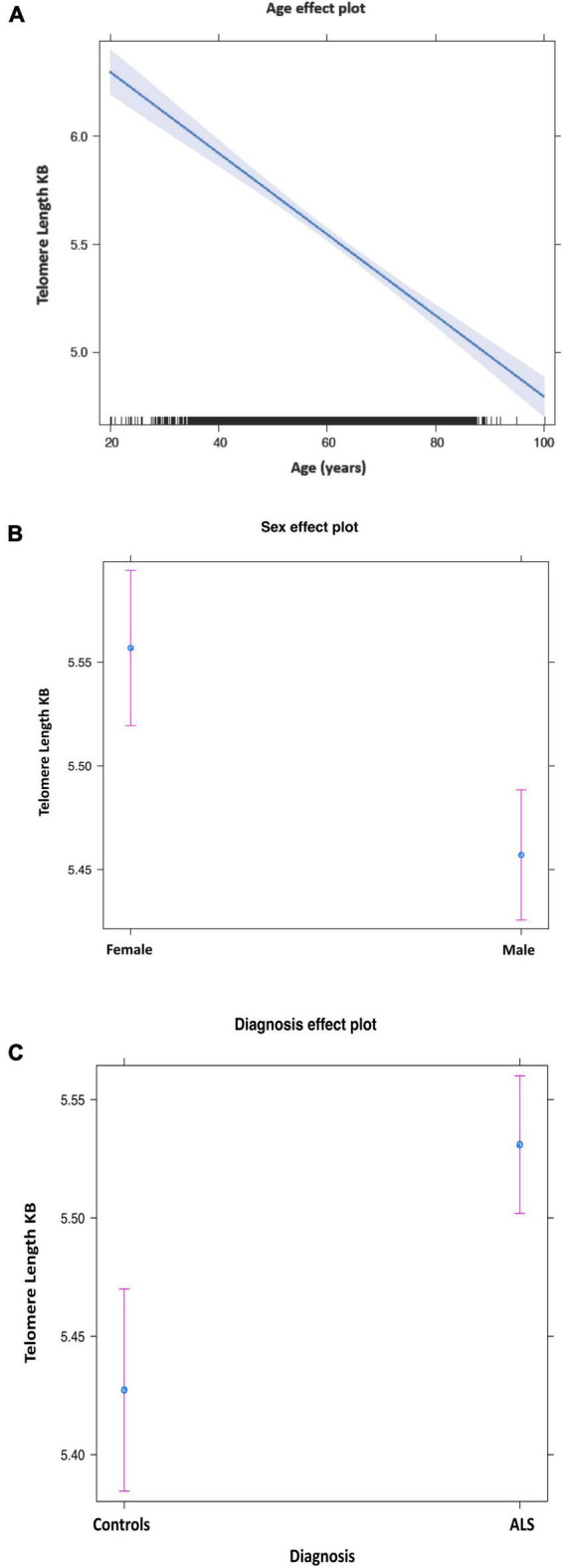
Mean telomere length by age **(A)**, sex **(B)**, and disease status **(C)**. Purple bars indicate 95% confidence intervals.

The mean telomere length in people with ALS was 5.5 kb, and in controls, 5.38 kb (Figure 1). Multivariable linear regression accounting for sex and age as covariates showed a mean 20% (95% CI 14, 25%) longer telomere length in people with ALS compared to controls (*p* = 1.1 × 10^–12^). Covariate analysis showed that regardless of disease status, females (*p* = 2.42 × 10^–5^) and younger people (*p* = 1.2 × 10^–16^) had on average longer telomeres (Table 2), confirming the results of earlier studies that telomere length reduces with age and females have on average longer telomeres.

**TABLE 2:** Telomere length comparison between people with amyotrophic lateral sclerosis (ALS) and healthy controls using a generalized linear model.

	Estimate	SD of estimate	*P*-value
Age (per year)	−2%	0.02	1.1 × 10^–16^
Sex (male vs. female)	−8%	0.03	2.42 × 10^–5^
Case-control status (controls vs. cases)	−29%	0.02	1.1 × 10^–12^

To assess if the observed longer telomere length in apparently sporadic ALS is also seen in familial ALS, we assessed telomere length in 79 people, not included in the main analysis, with a family history of ALS in a first degree relative (FALS). Multivariable linear regression after correcting for age and sex again showed a longer average telomere length in people with FALS than in controls (*p* = 2.0 × 10^–16^).

Examining the effect of age on telomere length in ALS and controls separately, showed that the rate of shortening by age is slower in ALS than in controls, suggesting it is not an active lengthening of telomeres in ALS (0.022% per year, *p* < 0.0001 vs. 0.012% per year, *p* < 0.0001) and also arguing against the possibility that telomeres are longer to start with in people who will later develop ALS. The rate of shortening was different in males and females, with females showing a faster rate of shortening than males (Supplementary Figure 1).

In an analysis exploring survival bias as an explanation for our results, we restricted testing to those younger than the median age (62 years). Multivariable linear regression accounting for sex and age still showed that telomeres were longer in people with ALS compared to controls (*p* = 8.12 × 10^–12^) with mean telomere length in people with ALS, 5.8 kb, and in controls, 5.5 kb. To ensure that telomere length analysis was not biased by population effects, we excluded the UK, a population we used previously for discovery analysis, and using a subset of samples the association was still observed (*p* = 6.6 × 10^–9^).

**FIGURE 2:**
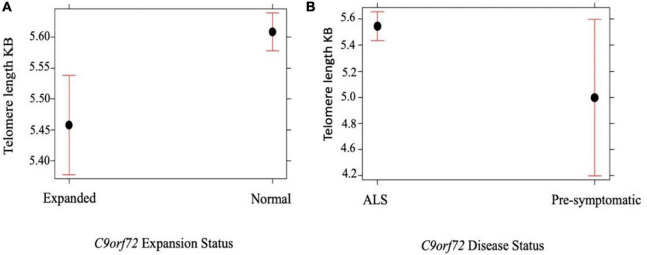
**(A)** Telomere length comparison between 334 people with ALS carrying an expanded *C9orf72* repeat against people with ALS not carrying an expansion. Multivariable linear regression shows that the telomere is shorter in those carrying a *C9orf72* expansion compared with age and sex matched disease controls not carrying an expansion (*p* = 5.0 × 10^– 4^). **(B)** Telomere length comparison between 334 people with ALS with *C9orf72* repeat expansion against 10 healthy individuals with confirmed expanded *C9orf72* status. Multivariable linear regression shows that the telomere is longer in people with ALS with *C9orf72* repeat expansion (*p* = 0.05). Telomere length reported in kilobases (kb). Red bars indicate 95% confidence intervals.

We compared telomere length in 334 people with ALS with *C9orf72* repeat expansion against people with ALS with confirmed non-expanded *C9orf72* status. Multivariable linear regression showed that the telomere was shorter in expansion carriers (*p* = 5.0 × 10^–4^) (Figure 2A and Table 3). Although ALS *C9orf72* expansion carriers had a shorter telomere length than non-expansion carriers, telomere length was still longer in those carrying a *C9orf72* repeat expansion than controls (*p* = 0.001) (Figure 2B).

**TABLE 3:** Telomere length comparison between 552 people with amyotrophic lateral sclerosis (ALS) with a *C9orf72* repeat expansion and 907 people with ALS with normal *C9orf72* repeat length using a multivariable linear regression.

	Estimate	SD of estimate	*P*-value
Age (per year)	−2%	0.02	1.1 × 10^–16^
Sex (male vs. female)	−15%	0.03	2.42 × 10^–5^
*C9orf72* (expanded)	−27%	0.02	5.0 × 10^–4^

We therefore assessed the relationship between the estimated number of telomere repeats and estimated number of *C9orf72* repeats using ExpansionHunter in 1,589 samples from the UK. Multivariable linear regression showed that the number of telomere repeats is associated negatively with *C9orf72* repeat expansion size (*p* = 0.003) supporting the previous results.

To assess if the relationship between *C9orf72* repeat expansion and telomere repeat size was specific to *C9orf72*-mediated ALS, we ran ExpansionHunter on three ALS genes which also contain disease-associated repeat expansions: *ATXN1*, *ATXN2*, and *NIPA1*. There was no difference in telomere length observed between expansion carriers and non-expansion carriers for any of these genes.

Cox regression analysis showed that people with ALS with telomere length less than 5.3 Kb had a 10% increase in median survival compared with those with longer telomeres (*p* = 5.0 × 10^–7^) after correcting for age, sex, site of onset, *C9orf72* status and principal components of ancestry.

To validate our findings in a different tissue we used 159 *post-mortem* brain samples, 106 from people with apparently sporadic ALS and 53 controls. The male-female ratio was 2:1. The mean telomere length in people with ALS was 6.8 kb, and in controls, 6.56 Kb, not taking into account gender or age. Multivariable linear regression accounting for these covariates showed that telomere length in people with ALS was longer by mean 29% (95% CI 30, 55%) compared with controls (*p* = 0.03).

## Discussion

Using a large disease-specific whole genome sequencing dataset, we have shown that longer telomeres are associated with ALS, confirming initial findings from a pilot study (Al Khleifat et al., 2019). We were additionally able to show that our findings are likely a result of less rapid shortening of telomeres being associated with ALS, rather than active telomere lengthening in ALS. In keeping with expectations, we also found that mean telomere length was on average longer in females, and in all samples, shortened with increasing age. The association of longer telomeres with apparently sporadic ALS was also seen in FALS, supporting the notion that familial and sporadic ALS are not mutually exclusive categories but rather a spectrum (Al-Chalabi and Hardiman, 2013; Al-Chalabi et al., 2014; Chiò et al., 2018; Mehta et al., 2018). Furthermore, telomere length was inversely correlated with *C9orf72* repeat expansion size.

Telomere elongation phenomena are well-documented but far less well-understood than telomere shortening phenomena (Bryan et al., 1995; Cesare and Reddel, 2010; Arora and Azzalin, 2015; Haycock et al., 2017). While telomere shortening is typically seen in cancers, telomere elongation can occur in cancers of the nervous system, and for example, is seen in 25% of primary brain tumors, in glioblastoma multiforme and in 10% of neuroblastomas (Bryan et al., 1997; Hakin-Smith et al., 2003; Henson et al., 2005; Durant, 2012; Boutou et al., 2013). In general, cancers in which cells have long telomeres are resistant to therapy and carry a poor prognosis (Haycock et al., 2017). Telomere elongation has also been associated with schizophrenia (Nieratschker et al., 2013; Zhang et al., 2018), a disorder that genetically overlaps with ALS (McLaughlin et al., 2017). Additionally, longer telomeres are also reported in Parkinson’s disease and Lewy body dementia blood and brain (Asghar et al., 2022).

We found that pathologically expanded *C9orf72* repeats are negatively associated with telomere repeat length, so that people with expanded *C9orf72* repeats had shorter telomeres on average. *C9orf72* gene repeat expansion is the most frequent genetic cause of ALS and of the related condition, frontotemporal dementia (Shatunov et al., 2010; Smith et al., 2013; Hardiman et al., 2017; Iacoangeli et al., 2019). A possible explanation for the negative association is in the liability threshold model of disease. Those people who already have a high liability to ALS do not need the additional liability of longer telomeres and so on average would appear to have shorter telomeres. Alternatively, those in the higher risk group (non-repeat expansion carriers) need a greater contribution from other sources of disease liability and so have longer telomere repeats. Against this explanation is the observation that other gene variants that predispose to ALS risk such as intermediate *ATXN2* repeat expansions, do not show any association with telomere length, and neither do people with familial ALS. The explanation might therefore lie in the *C9orf72* repeat expansion itself. Both telomeres and large *C9orf72* repeats have a tendency to fold into structures called G quadruplexes (Fratta et al., 2012; Grigg et al., 2014). G quadruplex structures have important roles in DNA replication, recombination and telomere maintenance (Millevoi et al., 2012; Bryan, 2019). Although there have been several studies of the G quadruplexes formed by *C9orf72* repeat expansion, the relationship between *C9orf72* repeats and other G quadruplexes such as telomeres is not documented at population level and has not been well-characterized *in vivo* (Zhang et al., 2019).

Some genetic variations that contribute to ALS risk also worsen prognosis. This is seen in carriers of the *C9orf72* repeat expansion mutation, those carrying the *UNC13A* homozygous risk genotype, and for some variants of the *SOD1* gene for example. In keeping with that pattern, we have found that longer telomere length is associated with ALS risk as well as with worse prognosis, with those with the shortest telomeres having a 10% increase in survival.

The genetic landscape of ALS is one of some monogenic causes, several gene variations that substantially but not dramatically increase risk, and a polygenic component. For those with a monogenic basis of their disease, the variable and age-dependent penetrance seen is likely because of the contribution of other factors to risk (Al-Chalabi and Hardiman, 2013; Al-Chalabi et al., 2014; Chiò et al., 2018; Garton et al., 2021). Based on these findings, telomere length could be such a factor.

The main limitation of this study is that we did not directly measure telomere length using Southern blotting, but estimated it using whole genome sequence data. The method we have used, TelSeq, was recently used to estimate telomere length in 75,000 whole genome sequences. Comparing the performance of TelSeq with other bioinformatics tools such as Computel, the estimates of telomere length were highly correlated between the bioinformatics methods and with Southern blot results, with the advantage that TelSeq had a faster processing time (Taub et al., 2019), although it is not possible to draw conclusions about the exact length of a telomere. Other studies have also shown a good correlation between TelSeq telomere length estimates, Southern blotting and Q-PCR (Ding et al., 2014; Cook et al., 2016). With this in mind, different sequencing technologies might generate different telomere length estimates because of differences in library preparation and platform (Ma, 1995; Aviv et al., 2011). To overcome this potential weakness, we have used the same industry-leading sequencing platform for all samples, as well as designing the study to minimize batch effects by having cases and controls sharing the same sequencing plate. Our study has the advantage of a large sample size of more than 4,500 cases, far larger than for previous reports.

We have shown that despite being an age-related, male-predominant condition, ALS is associated with longer telomere lengths in blood-derived and in brain-derived DNA.

## Data availability statement

The data presented in this study are deposited in the Project MinE consortium public repository (http://databrowser.projectmine.com).

## Ethics statement

The studies involving human participants were reviewed and approved by the Research Ethics Board at each respective recruiting site within the Project MinE consortium as previously described (van Rheenen et al., 2018).

## Author contributions

AA-C and AAK conceived and planned the study, wrote the first draft of the manuscript, did the statistical analysis, and prepared the figures and tables. AAK and AI created the bioinformatics pipeline for analysis. JvV ran ExpansionHunter on Project MinE data. MM and RZ prepared phenotypic data. AA-C, JHV, OH, MP, JM, PS, JL, CS, NB, OH, WR, PVD, and LB helped in sample collection and provided whole genome sequence data and analysis and intellectual input for data interpretation on behalf of the Project MinE Consortium. JHV, OH, and MM provided intellectual input for data interpretation. All authors reviewed and approved the final manuscript.
