# Supplementary material for: Telomere length analysis in amyotrophic lateral sclerosis using large-scale whole genome sequence data
Source: Front Cell Neurosci. 2022 Dec 15;16:1050596. doi: 10.3389/fncel.2022.1050596 (PMC9799999; doi:10.3389/fncel.2022.1050596)
Supplement: Supplementary file 1 [file Data_Sheet_1.pdf]

Supplementary Material

Quality Control

Quality control was performed separately on genotyped data of each population according to the Project MinE methods published previously[1]. After quality control, the full set of genomic Variant Call Format files (gVCFs) were merged together by first converting the gVCFs to Plink format and then merging all files together. This generated a single dataset containing all variant sites across all individuals. Non-autosomal chromosome and multi-allelic variants were excluded from pilot analyses. Sample and SNP quality control were performed using Plink[1,2] and VCFtools[3]. To begin sample quality control, missingness by sample was calculated on a per-chromosome basis.

All other sample quality control steps were performed on a set of high-quality biallelic SNPs that had minor allele frequency at least 10%, missingness < 0·1%, were linkage disequilibrium pruned at an r^2 threshold of 0·2, were not A/T or C/G SNPs, did not lie in the major histocompatibility complex or lactase gene locus, and did not occur in the inversions on chromosome 8 or chromosome 17. The ~30,000 SNPs overlapping this set of SNPs and HapMap 3 were used to calculate principal components projecting the ALS cases and controls onto the HapMap 3 samples. Samples of non-European ancestry, defined as further than 10 standard deviations from the European-ancestry population principal components in HapMap 3 (CEU, people of Northern and Western European ancestry living in Utah; TSI, Tuscans in Italy), were excluded from analysis to ensure an ancestrally homogeneous group of samples for association testing. Samples with an inbreeding coefficient > 3 standard deviations from the mean of the distribution were excluded, as were unexpectedly related samples. Genotypes available from genotyping on the Illumina Omni 2.5M array were compared to sequencing genotypes, and samples with < 95% concordance were dropped from the analysis. Lastly, samples with discordant sex information (comparing chromosome X genotypes and phenotype information) were excluded.

For variant quality control, variants with missingness > 5% were removed, as were variants out of Hardy-Weinberg equilibrium in controls ($p < 1 \times 10^{-6}$). Differential missingness between cases and controls was checked and variants with $p < 1 \times 10^{-6}$ were removed. Variants with extreme low or extreme high depth of coverage (> 6 standard deviations from the mean of the total depth distribution) were also excluded. Finally, the mitochondrial, X and Y chromosomes were excluded from analysis (but will be included in later analyses as sample sizes in Project MinE continue to grow). Approximately 10 million sites were lost during variant quality control.

References:

1. van Rheenen, W. *et al.* Genome-wide association analyses identify new risk variants and the genetic architecture of amyotrophic lateral sclerosis. *Nat. Genet.* **48**, 1043–1048 (2016).

2. Purcell, S. *et al.* PLINK: A tool set for whole-genome association and population-based linkage analyses. *Am. J. Hum. Genet.* **81**, 559–575 (2007).

3. Danecek, P. *et al.* The variant call format and VCFtools. *Bioinformatics* (2011) doi:10.1093/bioinformatics/btr330.

Supplementary Figures

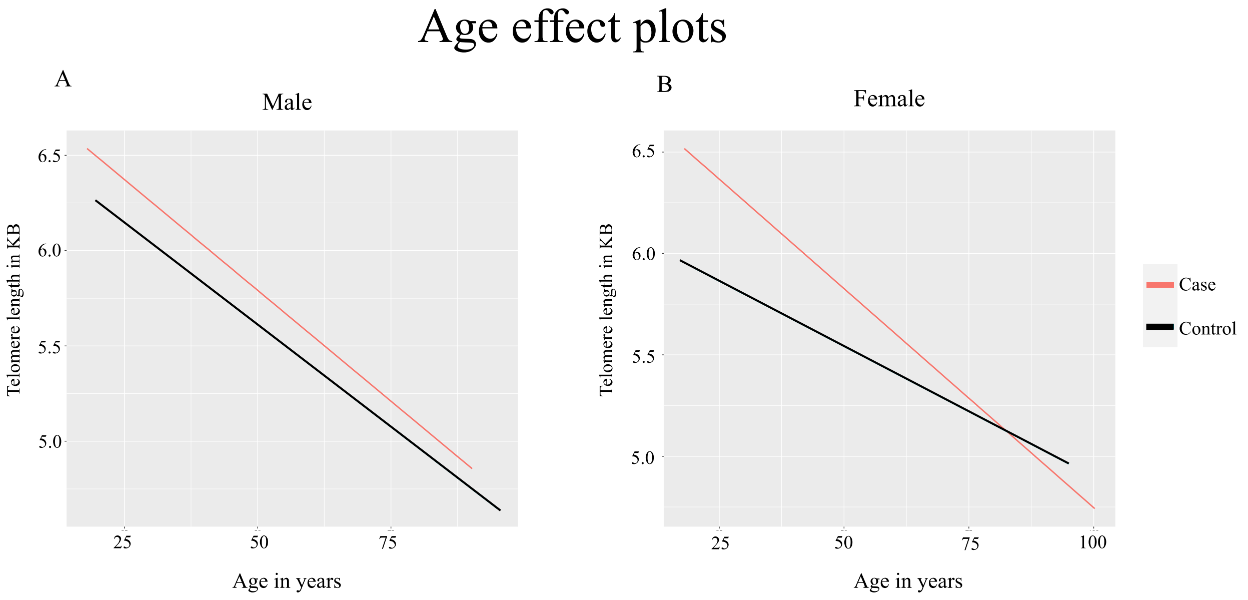

Supplementary Figure 1. Mean telomere length by age (A) Male, (B) Female. Red line indicates ALS cases and black line indicates controls.

Supplementary Tables

Supplementary table 1

	Estimate	SD of estimate	p=Value
**Age (per year)**	-2%	0.02	1.1×10^{-16}
**Sex (male vs. female)**	-8%	0.03	2.42×10^{-5}
**Case-control status (controls vs. cases)**	-29%	0.02	1.1×10^{-12}
**Technology (HiSeqX vs. HiSeq2500)**	5.00%	0.02	0.001
**PC1**	4.50%	1.44	0.001
**Country**	0.80%	0.05	0.12

Telomere length comparison between people with ALS and controls using a generalized linear model. PC1 The first principal component.

Supplementary table 2

	Estimate	SD of estimate	p=Value
**Age (per year)**	-2%	0.02	1.1×10^{-16}
**Sex (male vs. female)**	-15%	0.03	2.42×10^{-5}
***C9orf72* (expanded)**	-27%	0.02	5.0×10^{-4}
**Technology (HiSeqX vs. HiSeq2500)**	1.90%	0.12	0.07
**PC1**	-2.48%	2.55	0.33

Telomere length comparison between 552 people with ALS with a *C9orf72* repeat expansion and 907 people with ALS with normal *C9orf72* repeat length using a multivariable linear regression. PC1 The first principal component.
